# Prophylactic intravitreal injection of aflibercept for preventing postvitrectomy hemorrhage in proliferative diabetic retinopathy: A randomized controlled trial

**DOI:** 10.3389/fpubh.2022.1067670

**Published:** 2023-01-11

**Authors:** Jinfeng Qu, Xiuju Chen, Qinghuai Liu, Fang Wang, Mingxin Li, Qiong Zhou, Jin Yao, Xiaoxin Li

**Affiliations:** ^1^Department of Ophthalmology, Peking University People's Hospital, Beijing, China; ^2^Department of Ophthalmology, Xiamen Eye Center of Xiamen University, Xiamen, China; ^3^Department of Ophthalmology, Nanjing Medical University Affiliated Nanjing Hospital, Jiangsu, China; ^4^Department of Ophthalmology, The Tenth Affiliated Hospital of Shanghai Tongji University, Shanghai, China; ^5^Department of Ophthalmology, The Affiliated Hospital of Xuzhou Medical University, Jiangsu, China; ^6^Department of Ophthalmology, The First Affiliated Hospital of Nanchang University, Jiangxi, China; ^7^Department of Ophthalmology, The Affiliated Eye Hospital of Nanjing Medical University, Jiangsu, China

**Keywords:** aflibercept, vitreous hemorrhage, vitrectomy, diabetic retinopathy, post-vitrectomy hemorrhage

## Abstract

**Introduction:**

The aim of this study was to assess the effects of preoperative intravitreal aflibercept (IVA) injection on the incidence of postoperative vitreous hemorrhage (VH) after vitrectomy for proliferative diabetic retinopathy (PDR).

**Methods:**

This study involved a prospective, randomized clinical trial. One hundred twenty-eight eyes of 128 patients of PDR who underwent pars plana vitrectomy (PPV) were enrolled. Sixty-four eyes were assigned randomly to either the IVA group (IVA injection 1 to 5 days before PPV) or the control group (no IVA injection). The primary outcome was the incidence of VH at 1 month after PPV. Secondary outcome measures were best-corrected visual acuity (BCVA) changes from baseline to at 1 week, 1 month, 2 months, and 3 months after surgery.

**Results:**

The VH incidences in the IVA group and the control group were 14.8 and 39.3% at week 1, 8.6 and 31.7% at month 1, 11.7 and 30.5% at month 2, and 8.6 and 30.5% at month 3, respectively. Intergroup differences showed a significantly decreased VH rate in the IVA group compared with that in the control group at week 1, month 1, and month 3 (*p* = 0.021, 0.006, and 0.047, respectively). Compared to the baseline, neither the mean BCVA nor the BCVA change in the Logarithm of the Minimum Angle of Resolution (logMAR) scale did differ significantly between the two groups at each visit point. There are a greater number of eyes with BCVA improvement of more than 2 logMAR in the IVA group than in the control group at week 1 (8 vs. 2, *p* = 0.048).

**Conclusions:**

This study found that the adjunctive use of preoperative IVA reduces early and late postoperative VH in vitrectomy for PDR.

## Introduction

Patients with diabetic retinopathy (DR) are becoming more and more predominant in many countries with the increasing prevalence of diabetes worldwide. Diabetic retinopathy is the leading cause of vision loss in patients with diabetes. The standard and effective surgical treatment for vitreous hemorrhage (VH) and tractional retinal detachment (TRD) for proliferative diabetic retinopathy (PDR) is pars plana vitrectomy (PPV). Although the anatomical success rate of vitrectomy for PDR is good, it can have a few postoperative complications sometimes. Postvitrectomy VH is one of the most common complications after vitrectomy in PDR and has been reported with success rates ranging between 17 and 75% (1–6). Patients may have delayed visual rehabilitation because of postvitrectomy VH due to obscuration of the fundus, and postvitrectomy VH may hinder the monitoring of the disease course and/or create the need for additional application of laser treatment.

The level of vascular endothelial growth factor (VEGF) is elevated in the retina of patients with diabetes (7, 8). A previous study reported that a higher VEGF level in the vitreous humor during the primary vitrectomy could be a risk factor for early postoperative VH and neovascular glaucoma (NVG) in patients with PDR (9). Recently, many studies reported that the preoperative vitreous injection of an anti-VEGF agent can reduce VH after PPV for patients with PDR. Most studies reported that providing intravitreal bevacizumab (IVB) injection before PPV can increase the feasibility of PPV and reduce active retinal neovascularization, intraoperative bleeding, surgical time, and postoperative VH (10–21). These findings suggest that a high VEGF level at primary vitrectomy contributes to the development of postoperative VH for PDR.

Aflibercept is a fully humanized recombinant fusion protein that has a molecular weight of 115 kDa and is made by fusing the fragment crystallizable (Fc) region of human immunoglobulin G (IgG) to the second domain of vascular endothelial growth factor receptor 1 (VEGFR-1) and the third domain of vascular endothelial growth factor receptor 2 (VEGFR-2). It can bind to not only all subtypes of VEGF but also placental growth factor (PlGF). The affinity of aflibercept for vascular endothelial growth factor-A165 (VEGF-A165) is 94 times greater than that of ranibizumab and approximately 120 times greater than that of bevacizumab. The intravitreal half-life of aflibercept is greater than those of ranibizumab and bevacizumab (4.7 vs 2.9 days and 4.3 days) (22). It has been proven to be effective in inducing retinal neovascularization regression in patients with PDR, but a well-structured prospective study about the adjunctive use of intravitreal injection of aflibercept (IVA) to reduce postoperative VH in PPV for PDR is still lacking. In this study, we aimed to assess the effect of preoperative IVA on the incidence of postoperative VH after PPV for PDR.

## Methods

This was a prospective, randomized clinical trial (NCT 05478967). The study followed the tenets of the Declaration of Helsinki and was approved by the Institutional Review Board of the Peking University People's Hospital. Written informed consent was signed by all participants before enrollment.

The present study enrolled patients with PDR at the Department of Ophthalmology, Xiamen Eye Center of Xiamen University, between August 2019 and March 2021. The inclusion criteria were as follows: (1) patients with type 2 diabetes mellitus (T2DM), (2) patients aged between 35 and 65 years, and (3) patients with PDR who underwent primary pars plana vitrectomy (PPV) for VH. The following cases were excluded from analysis: (1) eyes with retinal tear, (2) eyes with iris or anterior angle neovascularization, (3) eyes with intraoperative use of silicone oil, (4) eyes with choroidal or retinal disease other than PDR or any inflammatory condition, (5) eyes that underwent any previous vitrectomy or scleral buckle surgery, (6) eyes that received intraocular triamcinolone acetonide (TA) injection within 90 days before screening, (7) eyes that received intraocular anti-VEGF treatment within 60 days before screening or contralateral eyes received intraocular anti-VEGF treatment during follow-up, (8) patients who had taken aspirin orally within 7 days before screening, (9) patients who had coagulation mechanism disorder or had taken any other medicine for anticoagulant treatment, (10) patients who had cerebrovascular accident and/or myocardial infarction occurring within 180 days before screening, (11) patients with uncontrolled blood pressure (sitting position > 160/100 mmHg), (12) patients with liver or kidney dysfunction or any severe systemic disease, and (13) patients who accepted any anti-VEGF therapy for the study eye during the follow-up. If both eyes of the same patients were eligible, the eye with worse vision was included in the study.

The enrolled eyes were randomly assigned, according to the Central Randomization System, with a ratio of 1:1 to the IVA group and the control group. Patients in the IVA group received an IVA (0.5 mg/0.05 ml) injection before surgery (1 to 5 days before surgery); patients in the control group did not receive IVA injection before vitrectomy. The preoperative IVA injection was given following a standard protocol. All patients underwent 25-gauge transconjunctival sutureless vitrectomy using the 25-gauge trocar and cannula system under local anesthesia. Procedures, such as fibrovascular membrane dissection, endodiathermy, or endolaser photocoagulation, were performed with 25-gauge instruments, as required. Pan-retinal photocoagulation (PRP) was complicated as much as possible during the surgery. Intraoperative bleeding was controlled either by endodiathermy or by increasing the irrigation pressure.

Patients were examined 1 week, 1 month, 2 months, and 3 months after surgery if there were no postoperative events. If postoperative complications, including VH, occurred, patients were instructed to visit the clinic, regardless of the visit schedule. At each visit, any events involving the study eye between the visit schedules were recorded accordingly. At each postoperative visit, slit-lamp biomicroscopy, indirect ophthalmoscopy, and fundus photography were performed.

The primary outcome was the incidence of VH at 1 month after PPV. Secondary outcome measures were best-corrected visual acuity (BCVA) changes from baseline at 1 week, 1 month, 2 months, and 3 months after surgery. Preoperative, intraoperative, and postoperative data were collected for each patient. Preoperative data included age, sex, duration, and status of diabetes mellitus [hemoglobin A1c (HbA1c)]; the presence of other systemic diseases such as hypertension and renal function (serum creatinine); and ophthalmic parameters including best-corrected visual acuity (BCVA), intraocular pressure (IOP), lens status, previous PRP, and indication for surgery. Intraoperative data included phacoemulsification and intraocular lens (IOL) procedures, sulfur hexafluoride (SF6) or air tamponade, and the presence of fibrovascular proliferation and tractional retinal detachment. Postoperative data included BCVA at each visit and the number of episodes of complications. Postoperative VH was defined as a new episode of VH of grade 1 or above, occurring later than 3 days after the primary surgery and was evaluated according to the Diabetic Retinopathy Vitrectomy Study grading system. Incidences of VH at week 1, month 1, month 2, and month 3 were recorded. In the case of a gas-injected eye, complications were assessed in the region without the gas bubble. Outcome assessors were masked from the allocation of each study eye.

The present study compared baseline clinical data and postvitrectomy complications between the IVA group and the control group in patients with PDR. The chi-square test and the Mann–Whitney test were used. A *P-*value of < 0.05 was considered statistically significant. All analyses were performed using SPSS 18.0.

## Results

One hundred fifty-four eyes were enrolled in the study and allocated randomly into two groups: 78 eyes in the IVA group and 76 eyes in the control group. During follow-up, 26 eyes were excluded: 16 cases because of the loss of follow-up and 10 eyes because of the administration of intravitreal silicone oil injection. For the final data analysis, 64 eyes in each group were included.

Patient demographics and preoperative clinical findings of the two groups are summarized in Table 1. A total of 128 eyes of 128 patients (82 males and 46 females) who underwent vitrectomy for PDR were studied. Their median age was 54 years (range, 29–86 years). The median HbA1c level was 6.7% (range, 3.9–23%). Fifty-two patients (43%) had hypertension and 7 patients (6.3%) had renal dysfunction. There were no statistically significant differences in age, gender, duration of diabetes mellitus (DM), hypertension, hemoglobin A1c (HbA1c), renal dysfunction, previous history of PRP, baseline BCVA, lens status, the severity of VH, and size and location of retinal proliferation between the two groups at baseline. The mean number of laser shots added during the surgery was 514.4 ± 260.3 in the IVA group and 581.7 ± 355.0 in the control group, and there was no statistically significant difference between them (*p* = 0.229).

**Table 1:** Patient demographics and preoperative clinical findings of the two groups.

		**Total**	**Aflibercept group**	**Control group**	***P* value**
Eyes (no.)		128	64	64	
Gender (no.)	Male	82	44	38	0.269
	Female	46	20	26	
Age (years)		54.3 ± 10.9	52.9 ± 10.5	55.3 ± 10.9	0.420
DM duration (years)		8.7 ± 5.1	8.4 ± 5.2	9.0 ± 5.0	0.503
HbA1c		6.7 ± 2.2	6.9 ± 2.8	6.5 ± 1.1	0.190
HTN	Yes	52	27	25	0.896
	No	69	35	34	
Renal dysfunction	Yes	7	5	2	0.438
	No	105	51	54	
Pre-op BCVA (LogMAR)		1.53 ± 0.81	1.49 ± 0.81	1.57 ± 0.80	0.590
IOP (mmHg)		14.8 ± 3.0	14.6 ± 2.9	14.9 ± 3.0	0.375
Lens status (no.)	Phakic	125	63	62	1.000
	Pseudophakic	3	1	2	
Previous PRP	No	48	29	19	0.110
	Partial	12	3	9	
	Complete	14	8	6	
	Cannot grade	54	24	30	
Pre-op VH grade	Mild (visible optic disc and large vessels)	30	15	15	0.268
	Moderate (only optic disc visible)	51	29	22	
	Severe (no view of the fundus)	37	16	21	
Pre-op tractional retinal detachment	No	57	29	28	0.637
	Yes	15	9	6	
	Cannot grade	56	26	30	
Location of proliferation	No	26	11	15	0.639
	Within vascular arcade	62	30	32	
	Involve equator	37	21	16	
	Beyond equator	3	2	1	
Size of proliferation	< 1 PD	52	22	30	0.279
	1~5 PD	58	33	25	
	>5 PD	16	7	9	

**Figure 1:**
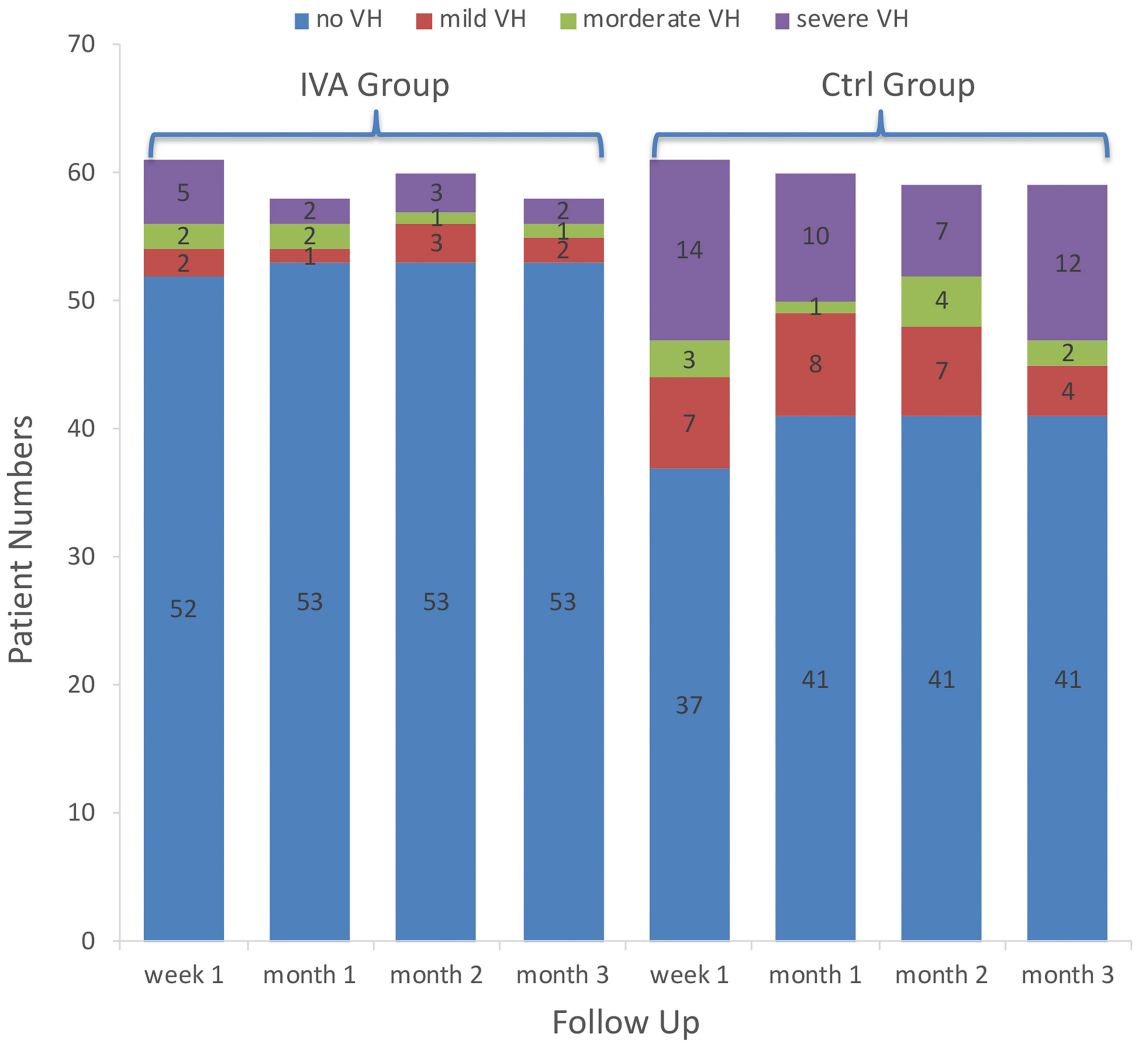
Vitreous hemorrhage grading in the two groups. The number of patients with different categories of vitreous hemorrhage grading in the preoperative intravitreal aflibercept (IVA) injection group and control group (bar with dotted pattern) during follow-up.

The VH incidences in the IVA group and the control group were 14.8 and 39.3% at week 1, 8.6 and 31.7% at month 1, 11.7 and 30.5% at month 2, and 8.6 and 30.5% at month 3, respectively. Intergroup differences showed a significantly decreased VH rate in the IVA group compared with that of the control group at week 1, month 1, and month 3 (*p* = 0.021, 0.006, and 0.047, respectively). The incidence of VH did not differ significantly between the two groups at month 2 (*p* = 0.089) (Figure 1).

**Figure 2:**
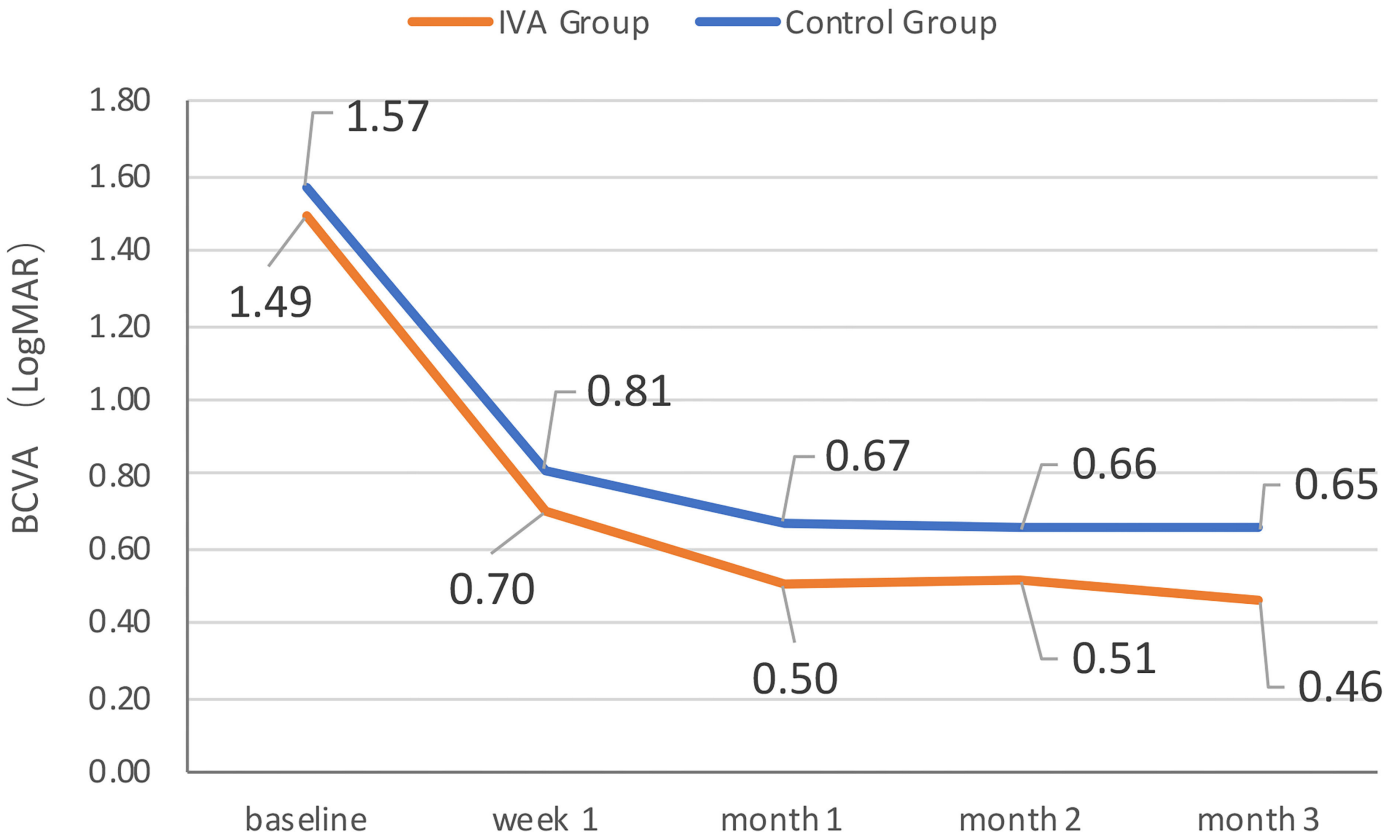
The mean best-corrected visual acuity (BCVA) at each visit in the two groups. The mean BCVA in logMAR (Logarithm of the Minimum Angle of Resolution) scale at baseline, week 1, month 1, month 2, and month 3 after surgery in the preoperative intravitreal aflibercept (IVA) injection group and control group.

**Figure 3:**
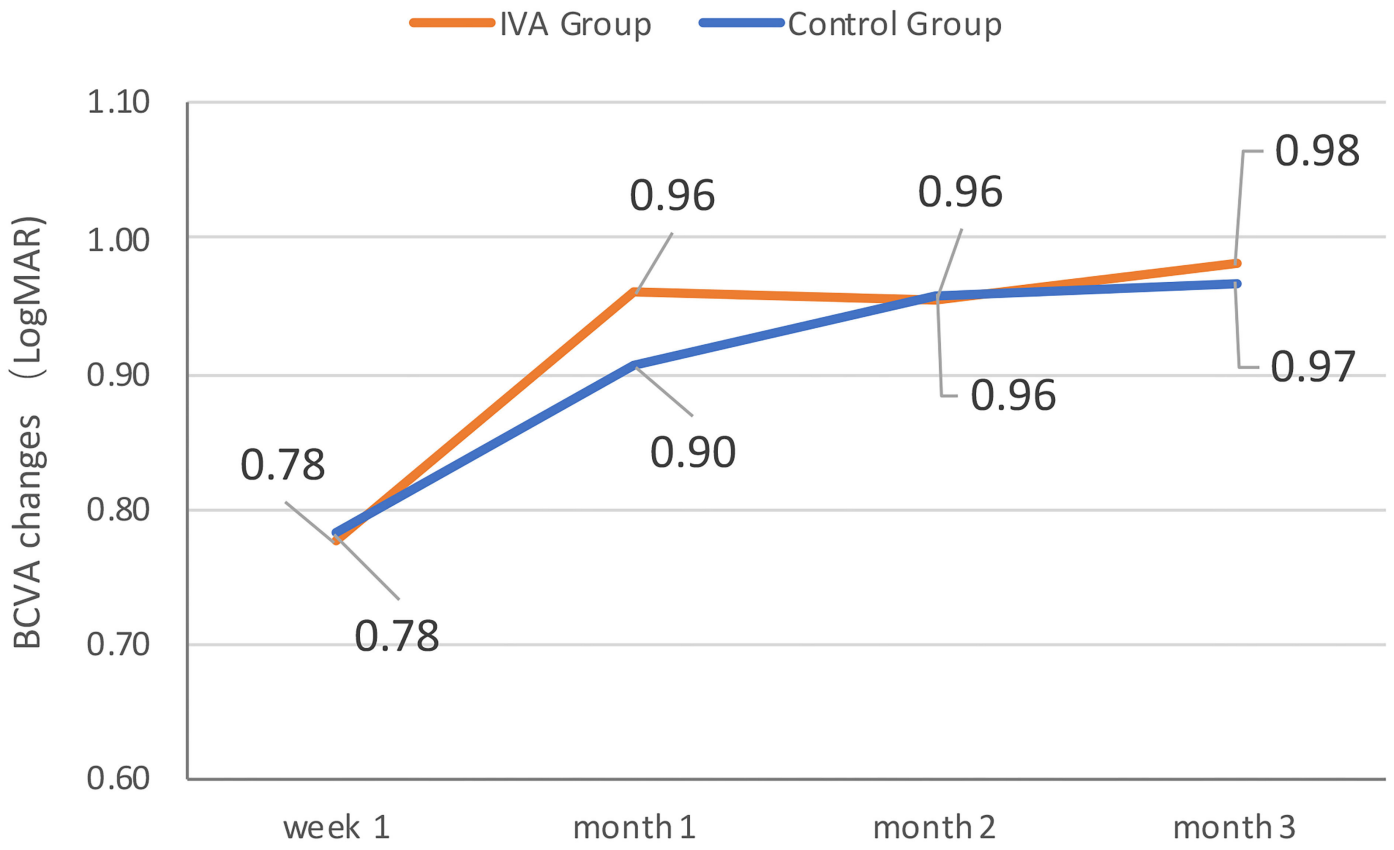
The mean best-corrected visual acuity (BCVA) change comparing to baseline during follow-up in the two groups. The mean BCVA change in logMAR (Logarithm of the Minimum Angle of Resolution) scale at week 1, month 1, month 2, and month 3 after surgery comparing to baseline in the preoperative intravitreal aflibercept (IVA) injection group and control group.

**Figure 4:**
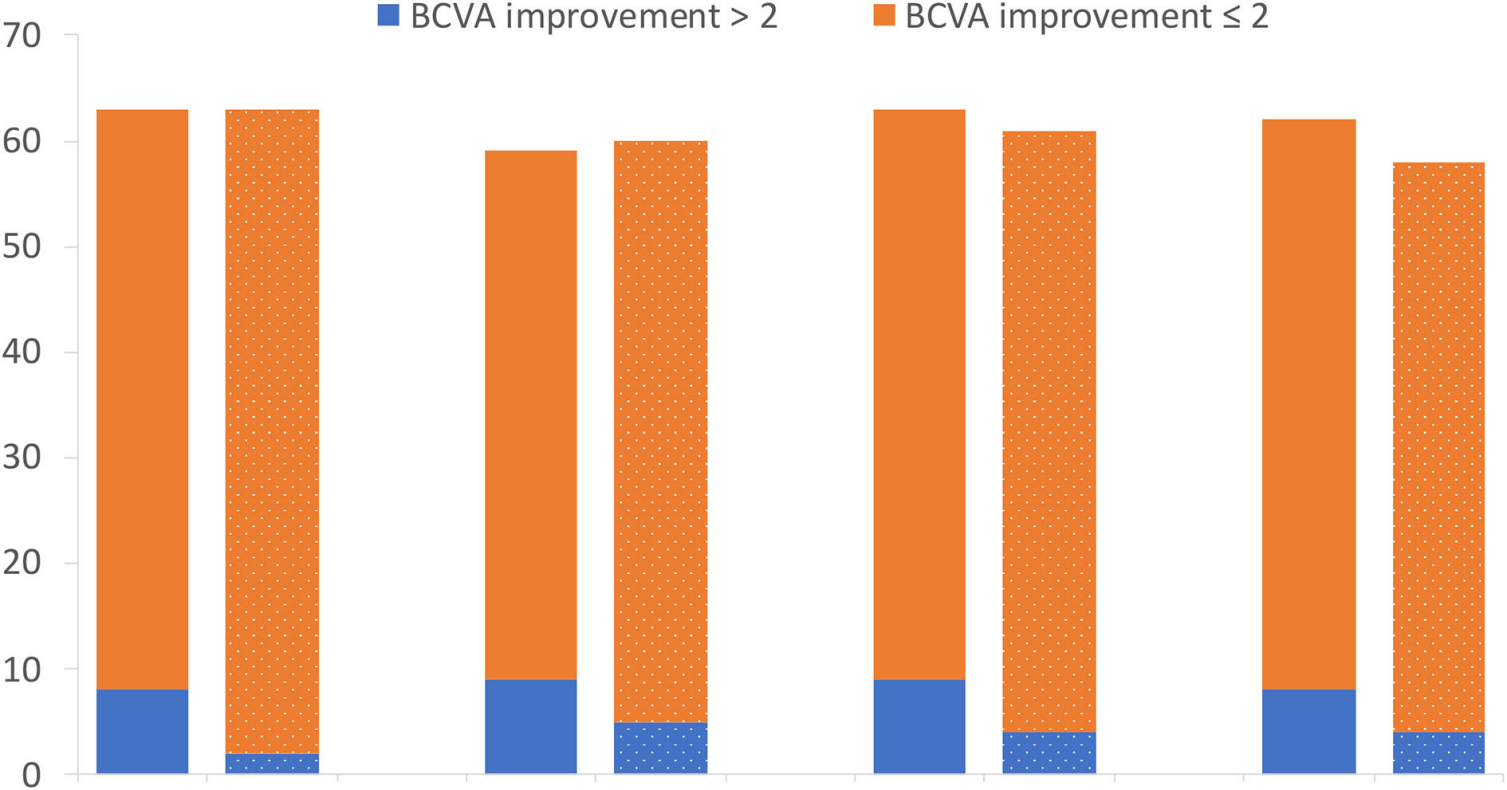
The number of patients with different categories of best-corrected visual acuity (BCVA) improvement in the two groups. The number of patients with different categories of BCVA improvement in logMAR (Logarithm of the Minimum Angle of Resolution) scale at week 1, month 1, month 2, and month 3 after surgery comparing to baseline in the preoperative intravitreal aflibercept (IVA) injection group and control group (bar with dotted pattern).

The mean BCVA changes in the logMAR scale were better in the IVA group than in the control group at week 1, month 1, month 2, and month 3 after surgery (0.70 vs. 0.81, 0.50 vs. 0.67, 0.51 vs. 0.66, and 0.46 vs. 0.65, respectively), but the difference between the two groups was not statistically significant at each visit point (*p* = 0.35, 0.11, 0.22, and 0.09, respectively) (Figure 2). The mean BCVA changes in the logMAR scale (baseline-visit) at week 1, month 1, month 2, and month 3 after surgery compared to baseline in the IVA group were 0.78, 0.96, 0.96, and 0.98, respectively. The mean logMAR BCVA changes at week 1, month 1, month 2, and month 3 after surgery compared to baseline in the control group were 0.78, 0.90, 0.96, and 0.97, respectively. The mean logMAR BCVA changes did not differ significantly between the two groups at each visit point (*p* = 0.84, 0.58, 0.81, and 0.73, respectively) (Figure 3). There are a greater number of eyes with BCVA change of more than 2 logMAR in the IVA group than in the control group at week 1 (8 vs. 2, *p* = 0.048), but this difference between the IVA group and the control group was not statistically significant at month 1, month 2, and month 3 after surgery (9 vs. 5, 9 vs. 4, and 8 vs. 4, respectively) (Figure 4).

There were no incidences of neovascular glaucoma (NVG), endophthalmitis, or TRD progression in the cases included for the final analysis.

## Discussion

Diabetic retinopathy (DR) is one of the leading causes of being legally blind in working-aged people and is responsible for up to 4.8% of blindness worldwide. Although PPV was the standard management for PDR with VH or TRD, they may suffer from postoperative VH. This complication will hinder fundus monitoring and/or additional retinal photocoagulation and delay their visual recovery.

The incidence of postoperative VH in PDR had been reported to be around 75% in the 1980s (1, 2), but with the development of surgical techniques and instruments, it has decreased to 12–40% in recent years. The risk factors of postoperative VH include younger age, later detection of DM, poor diabetic and hypertension control, higher serum creatinine, broader area of active neovascularization, increased extent of membrane peeling, postoperative hypotony, postoperative residual neovascularization membrane, unrelieved vitreous retinal contraction, and insufficient PRP (23, 24). In this study, baseline characters, including age, duration of DM, HbA1c, and patient proportion with renal dysfunction, were compared between the two groups, and vitrectomies were all performed by skillful surgeons with more than 15 years of experience to minimize the selective bias and heterogeneity in surgery.

Vascular endothelial growth factor has been proven to play an important role in the development of neovascularization in DR (7, 8). Preoperative vitreous injection of anti-VEGF may induce the regression of retinal neovascularization, decrease the intraoperative bleeding, make the dissection of fibrovascular membrane easier, and fasten vitreoretinal surgery. Previous studies showed that preoperative IVB can make vitrectomy easier and faster with less intraoperative bleeding. However, the effect of anti-VEGF intravitreal injection before vitrectomy on the incidence of postoperative recurrent VH had been reported in the literature with the controversial result. Ahmadieh et al. reported preoperative IVB was effective in reducing early (≤ 4 weeks) VH compared with a control group in patients with PDR (12). Ahn et al. compared preoperative IVB with intraoperative IVB and no IVB and found that the adjunctive use of IVB did not reduce postoperative VH incidence significantly (17). However, intraoperative IVB can significantly reduce early postoperative VH and fasten VH clearance compared with the control group. Lo et al. also found no significant differences in the postoperative VH rate between patients with PDR with and without preoperative IVB, but this study was limited by significant differences in the baseline characteristics between their groups (6).

These controversies exist because there are numerous differences in the detail of each strategy, such as the time point for the injection of anti-VEGF, the trocar gauge, and the suture of sclera wound. Heterogeneity of baseline characteristics among different studies also made it difficult to compare their results directly. Wang et al. conducted a network meta-analysis including 26 randomized controlled trials (RCTs) and 1,806 patients with PDR to compare preoperative anti-VEGF to the sham group. They found that injection at 6 to 14 days before vitrectomy could significantly reduce the duration of surgery, improve postoperative BCVA, and decrease the incidence of postoperative VH (25). Performing anti-VEGF injection at more than 14 days, 6 to 14 days, or 1 to 5 days before vitrectomy could significantly reduce the incidence of intraoperative bleeding, while there is no significant benefit for the incidence of postoperative VH. While in their studies, 19 RCTs used IVB, 4 used conbercept, and 1 used ranibizumab but none of them used aflibercept (25).

The ideal timing for pretreatment is controversial. Some authors suggested injecting more than 14 days before vitrectomy to make full use of anti-VEGF agents and induce the complete regression of neovascularization. However, Russo et al. studied the incidence of TRD following preoperative anti-VEGF injection and showed that the incidence of TRD after injection was 2.7% when the interval of injection and vitrectomy was less than 6 days, while it will be increased to 56% when the interval was prolonged to more than 10 days (25). A previous study showed that the timing of anti-VEGF therapy played an important role in the development of fibrosis, with longer lapses following IVB treatment resulting in increased levels of basic fibroblast growth factor (bFGF) (26). Another study showed that the expression of fibroblast cells and connective tissue growth factor (CTGF) increased in epiretinal fibrovascular membranes of the IVB group 21 days after treatment (27). Several investigators reported the progression to TRD after intravitreal injection of anti-VEGF in patients with PDR, which they call “anti-VEGF crunch syndrome” (28). It presents with the symptom of sudden vision loss after 1 to 6 weeks after intravitreal anti-VEGF injection in the affected eye. A higher dose of anti-VEGF may increase the severity of diabetic retinopathy and may be a risk factor for fibrosis. Tan et al. (28) reviewed these data and found that intravitreal anti-VEGF should be used with caution when treating patients with severe PDR and preexisting retinal fibrosis. They recommend close monitoring of crunch symptoms and proceeding promptly with surgery if there is new TRD or progression of TRD if patients underwent anti-VEGF injection before a planned vitrectomy. We injected the patients 1 to 5 days before vitrectomy in our study and did not find any formation or aggravation of TRD during the surgery.

Postoperative BCVA might be associated with numerous factors, such as the history of TRD, macular edema, macular ischemia, and ellipsoid zone (EZ) band integrity recurrent VH and gas or silicone oil tamponade (25). Zhao et al. did a meta-analysis including 14 RCTs involving 613 patients with PDR and found that patients in the anti-VEGF group achieved significantly better postoperative BCVA than those in the control group (29). Dervenis did another systemic review including 13 RCTs involving 688 patients with PDR and reported that preoperative IVB provided better long-term visual acuity (30). In another meta-analysis including more number of RCTs, they reported that performing only anti-VEGF injection given at 6 to 14 days before vitrectomy could significantly improve postoperative BCVA compared with the sham group (25). The present study found a greater number of eyes with BCVA improvement of more than 2 logMAR in the IVA group than in the control group at week 1, but this benefit disappeared in further follow-up and neither the mean BCVA nor the BCVA change did differ significantly between two groups. Whether this different result was related to the timing of preoperative injection or other factors cannot be concluded in this study.

To our knowledge, this is the first RCT that compares the incidence of postoperative VH between the preoperative IVA and sham groups for PDR. However, it has the following limitations: (1) failure to compare the influence of different timings of preoperative anti-VEGF injection; (2) having a relatively shorter follow-up period; and (3) the lack of a group with different anti-VEGF agents.

In conclusion, this RCT demonstrated that injective IVA at 1 to 5 days before vitrectomy for patients with PDR could reduce the incidence of early and late postoperative VH.

## Data availability statement

The raw data supporting the conclusions of this article will be made available by the authors, without undue reservation.

## Ethics statement

The studies involving human participants were reviewed and approved by Institutional Review Board of People's Hospital of Peking University. The patients/participants provided their written informed consent to participate in this study.

## Author contributions

JQ and XL contributed to the interpretation of the data. The first draft of the manuscript was written by JQ and all authors commented on previous versions of the manuscript. All authors contributed to the study's conception and design. All authors read and approved the final manuscript.

## References

[1] Schachat AP Oyakawa RT Michels RG Rice TA. Complications of vitreous surgery for diabetic retinopathy. II Postoperative complications. Ophthalmology. (1983) 90:522–30. 10.1016/S0161-6420(83)34540-1 6192378

[2] Novak M Rice T Michels R. Vitreous hemorrhage after vitrectomy for diabetic retinopathy. Ophthalmology. (1984) 91:1485–89. 10.1016/S0161-6420(84)34099-4 6521989

[3] West JF Gregor ZJ. Fibrovascular ingrowth and recurrent haemorrhage following diabetic vitrectomy. Br J Ophthalmol. (2000) 84:822–5. 10.1136/bjo.84.8.822 10906084 PMC1723593

[4] Mason JO III Nixon PA White MF. Intravitreal injection of bevacizumab (Avastin) as adjunctive treatment of proliferative diabetic retinopathy. Am J Ophthalmol. (2006) 142:685–8. 10.1016/j.ajo.2006.04.058 17011869

[5] Yang CM Yeh PT Yang CH. Intravitreal long-acting gas in the prevention of early postoperative vitreous hemorrhage in diabetic vitrectomy. Ophthalmology. (2007) 114:710–5. 10.1016/j.ophtha.2006.07.047 17275908

[6] Lo WR Kim SJ Aaberg TM Sr Bergstrom C Srivastava SK Marting DF. Visual outcomes and incidence of recurrent vitreous hemorrhage after vitrectomy in diabetic eyes pretreated with bevacizumab (Avastin). Retina. (2009) 29:926–31. 10.1097/IAE.0b013e3181a8eb88 19584650 PMC3033782

[7] Adamis AP Miller JW Bernal MT D'Amico DJ Folkman J Yeo TK. Increased vascular endothelial growth factor levels in the vitreous of eyes with proliferative diabetic retinopathy. Am J Ophthalmol. (1994) 118:445–50. 10.1016/S0002-9394(14)75794-0 7943121

[8] Loporchio DF Tam EK Cho J Chung J Jun GR Xia WM. Cytokine levels in human vitreous in proliferative diabetic retinopathy. Cells. (2021) 10:1069. 10.3390/cells10051069 33946446 PMC8147162

[9] Wakabayashi Y Usui Y Okunuki Y Ueda S Kimura K Muramatsu D. Intraocular VEGF level as a risk factor for postoperative complications after vitrectomy for proliferative diabetic retinopathy. Invest Ophthalmol Vis Sci. (2012) 53:6403–10. 10.1167/iovs.12-10367 22899753

[10] Rizzo S Genovesi-Ebert F Di Bartolo E Vento A Miniaci S Williams G. Injection of intravitreal bevacizumab (Avastin) as a preoperative adjunct before vitrectomy surgery in the treatment of severe proliferative diabetic retinopathy (PDR). Graefes Arch Clin Exp Ophthalmol. (2008) 246:837–42. 10.1007/s00417-008-0774-y 18286296

[11] Oshima Y Shima C Wakabayashi T Kusaka S Shiraga F Ohji M. Microincision vitrectomy surgery and intravitreal bevacizumab as a surgical adjunct to treat diabetic traction retinal detachment. Ophthalmology. (2009) 116:927–38. 10.1016/j.ophtha.2008.11.005 19269033

[12] Ahmadieh H Shoeibi N Entezari M Monshizadeh R. Intravitreal bevacizumab for prevention of early postvitrectomy hemorrhage in diabetic patients: a randomized clinical trial. Ophthalmology. (2009) 116:1943–8. 10.1016/j.ophtha.2009.07.001 19699531

[13] Arevalo JF Wu L Sanchez JG Monshizadeh R. Intravitreal bevacizumab (Avastin) for proliferative diabetic retinopathy: 6-months follow-up. Eye (Lond). (2009) 23:117–23. 10.1038/sj.eye.6702980 17891058

[14] Romano MR Gibran SK Marticorena J Wong D Heimann H. Can a preoperative bevacizumab injection prevent recurrent postvitrectomy diabetic vitreous haemorrhage? Eye (Lond). (2009) 23:1698–701. 10.1038/eye.2008.354 19039332

[15] Yeung L Liu L Wu WC Kuo YH Chao AN Chen KJ. Reducing the incidence of early postoperative vitreous haemorrhage by preoperative intravitreal bevacizumab in vitrectomy for diabetic tractional retinal detachment. Acta Ophthalmol. (2010) 88:635–40. 10.1111/j.1755-3768.2008.01498.x 19432872

[16] di Lauro R De Ruggiero P di Lauro R di Lauro MT Romano MR. Intravitreal bevacizumab for surgical treatment of severe proliferative diabetic retinopathy. Graefes Arch Clin Exp Ophthalmol. (2010) 248:785–91. 10.1007/s00417-010-1303-3 20135139

[17] Ahn J Woo SJ Chung H Park KH. The effect of adjunctive intravitreal bevacizumab for preventing postvitrectomy hemorrhage in proliferative diabetic retinopathy. Ophthalmology. (2011) 118:2218–26. 10.1016/j.ophtha.2011.03.036 21724263

[18] Smith JM Steel DH. Anti-vascular endothelial growth factor for prevention of postoperative vitreous cavity haemorrhage after vitrectomy for proliferative diabetic retinopathy. Cochrane Database Syst Rev. (2015) 7:CD008214. 10.1002/14651858.CD008214.pub3 26250103 PMC6599827

[19] Castillo J Aleman I Rush SW Rush RB. Preoperative bevacizumab administration in proliferative diabetic retinopathy patients undergoing vitrectomy: a randomized and controlled trial comparing interval variation. Am J Ophthalmol. (2017) 183:1–10. 10.1016/j.ajo.2017.08.013 28860046

[20] Tsubota K Usui Y Wakabayashi Y Suzuki J Ueda S Goto H. Effectiveness of prophylactic intravitreal bevacizumab injection to proliferative diabetic retinopathy patients with elevated preoperative intraocular VEGF in preventing complications after vitrectomy. Clin Ophthalmol. (2019) 13:1063–70. 10.2147/OPTH.S203921 31303746 PMC6605036

[21] Arevalo JF Lasave AF Kozak I Al Rashaed S Maia M Farah ME. Preoperative bevacizumab for tractional retinal detachment in proliferative diabetic retinopathy: a prospective randomized clinical trial. Am J Ophthalmol. (2019) 207:279–87. 10.1016/j.ajo.2019.05.007 31095954

[22] Balaratnasingam C Dhrami-Gavazi E McCann JT Ghadiali Q Freund KB. Aflibercept: a review of its use in the treatment of choroidal neovascularization due to age-related macular degeneration. Clin Ophthalmol. (2015) 9:2355–71. 10.2147/OPTH.S80040 26719668 PMC4689264

[23] Ding YH Yao BT Hang H Ye H. Multiple factors in the prediction of risk of recurrent vitreous haemorrhage after sutureless vitrectomy for non-clearing vitreous haemorrhage in patients with diabetic retinopathy. BMC Ophthalmol. (2020) 20:292. 10.1186/s12886-020-01532-8 32677996 PMC7367221

[24] Kameda Y Saeki T Hanai K Suzuki Y Uchigata Y Babazono T. Is chronic kidney disease affecting the postoperative complications of vitrectomy for proliferative diabetic retinopathy? J Clin Med. (2021)10:5309. 10.3390/jcm10225309 34830589 PMC8621452

[25] Wang DY Zhao XY Zhang WF Meng LH Chen YX. Perioperative anti-vascular endothelial growth factor agents treatment in patients undergoing vitrectomy for complicated proliferative diabetic retinopathy: a network meta-analysis. Sci Rep. (2020) 10:18880. 10.1038/s41598-020-75896-8 33144606 PMC7641141

[26] Li JK Wei F Jin XH Dai YM Cui HS Li YM. Changes in vitreous VEGF, bFGF and fibrosis in proliferative diabetic retinopathy after intravitreal bevacizumab. Int J Ophthalmol. (2015) 8:1202–6. 10.3980/j.issn.2222-3959.2015.06.22 26682173 PMC4651889

[27] Feng J Li B Wen J Jiang Y. Preoperative timing of intravitreal bevacizumab injection for proliferative diabetic retinopathy patients. Ophthalmic Res. (2018) 60:250–57. 10.1159/000493640 30380554

[28] Tan Y Fukutomi A Sun MT Durkin S Gilhotra J Chan WO. Anti-VEGF crunch syndrome in proliferative diabetic retinopathy: A review. Surv Ophthalmol. (2021) 66:926–32. 10.1016/j.survophthal.2021.03.001 33705807

[29] Zhao XY Xia S Chen YX. Antivascular endothelial growth factor agents pretreatment before vitrectomy for complicated proliferative diabetic retinopathy: a meta-analysis of randomised controlled trials. Br J Ophthalmol. (2018) 102:1077–85. 10.1136/bjophthalmol-2017-311344 29246890 PMC6059039

[30] Dervenis P Dervenis N Steel D Sandinha T Tranos P Vasilakis P. Intravitreal bevacizumab prior to vitrectomy for proliferative diabetic retinopathy: a systematic review. Ther Adv Ophthalmol. (2021) 13:25158414211059256. 10.1177/25158414211059256 34901749 PMC8655445

